# A Human Anti-c-Met Fab Fragment Conjugated with Doxorubicin as Targeted Chemotherapy for Hepatocellular Carcinoma

**DOI:** 10.1371/journal.pone.0063093

**Published:** 2013-05-13

**Authors:** Ximin Chen, Guipeng Ding, Qihe Gao, Jian Sun, Qianqian Zhang, Lijian Du, Zhenning Qiu, Changjun Wang, Feng Zheng, Bowang Sun, Jian Ni, Zhenqing Feng, Jin Zhu

**Affiliations:** 1 Key Laboratory of Antibody Technique of Ministry of Health, Department of Pathology, Nanjing Medical University, Nanjing, China; 2 Huadong Medical Institute of Biotechniques, Nanjing, China; 3 School of Chemistry and Chemical Engineering, Southeast University, Nanjing, China; 4 The Second Affiliated Hospital of Nanjing Medical University, Nanjing, China; 5 Jiangsu Key Laboratory of Cancer Biomarkers, Prevention & Treatment, Cancer Center, Nanjing Medical University, Nanjing, China; Institute of Molecular and Cell Biology, Singapore

## Abstract

c-Met is over-expressed in hepatocellular carcinoma(HCC) but is absent or expressed at low levels in normal tissues. Therefore we generated a novel conjugate of a human anti-c-Met Fab fragment (MetFab) with doxorubicin (DOX) and assessed whether it had targeted antitumor activity against HCC and reduced the side-effects of DOX. The MetFab was screened from human phage library, conjugated with DOX via chemical synthesis, and the conjugation MetFab-DOX was confirmed by HPLC. The drug release patterns, the binding efficacy, and cellular distribution of MetFab-DOX were assessed. MetFab-DOX was stable at pH7.2 PBS while release doxorubicin quickly at pH4.0, the binding efficacy of MetFab-DOX was similarly as MetFab, and the cellular distribution of the MetFab-DOX is distinct from free DOX. The cytotoxicity of MetFab-DOX was analyzed by the MTT method and the nude mouse HCC model. The MetFab-DOX demonstrated cytotoxic effects on c-Met expressing-tumor cells, but not on the cells without c-Met expression. MetFab-DOX exerted anti-tumor effect and significantly reduced the side effect of free DOX in mice model. Furthermore, the localization of conjugate was confirmed by immunofluorescence staining of tumor tissue sections and optical tumor imaging, respectively, and the tissue-distribution of drug was compared between free DOX and MetFab-DOX treatment by spectrofluorometer. MetFab-DOX can localize to the tumor tissue, and the concentration of doxorubicin in the tumor was higher after MetFab-DOX administration than after DOX administration. In summary, MetFab-DOX can target c-Met expressing HCC cells effectively and have obvious antitumor activity with decreased side-effects in preclinical models of HCC.

## Introduction

Hepatocellular carcinoma (HCC) is the sixth most common tumor worldwide, but due to its poor prognosis, it ranks as the third most common cause of death from cancer [1]. The major histological subtype of primary liver cancers, accounting for 70% to 85%, is hepatocellular carcinoma (HCC) [2]. The treatment of HCC includes hepatic resection, chemotherapy, radiotherapy, and so on, among which, the most effective is the surgical removal of the tumor tissue in the early stage of the HCC development [3], [4]. Unfortunately, when HCC is diagnosed, most of them are in the middle or late stage of the tumor progression, and the aforementioned therapies cannot work efficiently. Thus, it is necessary for us to develop novel effective therapies for treating HCC [5].

A major problem in HCC therapy is the lack of antitumor drugs with selectivity, so side effects to the normal tissues can not be avoided. One approach to enhance the specificity of the antitumor drugs is linking them to a carrier that can be preferentially taken up by tumor cells. Many carriers can be potential candidates for this purpose such as hormones, antibodies and liposomes. Among those methods, antibody-mediated tumor therapy has been developed lately. Cell-killing payloads such as protein toxins [6], radionuclides [7]–[9], and anticancer drugs [10]–[12] have been conjugated to monoclonal antibodies (mAbs) to generate immunotoxins, radioimmunoconjugates, and antibody-drug conjugates (ADCs), respectively, for tumor therapy. Among those methods, ADCs can transfer chemotherapy agents to the tumor cells directly by virtue of the specificity of the antibody against a molecule on the surface of the cells [13], [14]. Consequently, fewer side-effects as a result of chemotherapy can develop. Therefore, recent success has been achieved in mAb-targeted tumor therapy, and some ADCs have shown pronounced activities in preclinical models and are advancing toward or have entered clinical trials [15]–[20]. And an ADC (brentuximab vedotin) has been approved by FDA recently [21].

Through the Human Genome Project [22], [23],many proteins have been identified as molecular markers of liver tumor, such as α-fetoproteins, melanoma-associated antigens, and matrix metalloproteinases[24] Some of them have already been developed as molecular targets for cancer diagnosis and therapeutics. However, the current diagnostic accuracy and therapy efficacy for HCC are still far from satisfactory. Therefore, there is a great need to identify some new HCC-specific markers for more precise diagnosis and efficacious therapy of liver cancer. c-Met, the receptor of hepatocyte growth factor (HGF) that mediates a variety of biological activities, is important in the development and progression of various types of tumors, including HCC [25]–[28]. In tumor cells, c-Met activation mediated by HGF causes the triggering of a diverse series of signaling cascades resulting in cell growth, proliferation, invasion, and protection from apoptosis. c-Met transcription is increased in 30–100% of tumors compared to surrounding liver tissue. Similarly, c-Met is over-expressed at the protein level in 25–100% of HCCs compared to normal liver [29], suggesting a potential tumor-promoting role in HCC. Because of its over-expression in HCC but absent or expressed at low levels in normal tissues, c-Met has emerged as a promising drug target of personalized treatment for the HCC. Targeting the HGF/c-MET pathway in HCC has been reported. For example, three oral small molecule c-MET TKIs have demonstrated acceptable toxicity and modest clinical efficacy in Phase II trials in advanced HCC: foretinib [30], cabozantinib [31], and tivantinib [32].

Antibodies against c-met have been previously studied by our research group, including a murine anti-c-Met antibody as a multipurpose molecular diagnostics reagent [33], a human anti-c-Met Fab fragment and scFv fragment screening from human naive Fab library [27], [34]. We want to develop a serial of methods to apply anti-c-Met antibodies in the clinic tumor therapy, for example, developing antibodies that can block the HGF/c-Met pathway, conjugating antibodies with immunotoxins, radioimmunoconjugates and chemotherapy drugs. Some work has been published or under the way [35].

Doxorubicin (DOX) that exerts its cytotoxic activity by inhibiting the synthesis of nucleic acids within cancer cells [36], is a drug commonly used in cancer (e.g. HCC) chemotherapy with definite effects, while doxorubicin can cause serious side effects, especially heart and kidney toxicity, and myelosuppression [37]. As a result, DOX nowadays has limited clinical use in the patients of HCC. However, many investigators have still focused on this conventional chemotherapy agent to overcome the side-effect in the treatment of HCC. These mainly include two ways: new drug delivery systems, such as encapsulation of liposome [38], conjugation of PEG [39] or sulfonated aluminum phthalocyanine [40] to DOX. new route of administration, such as transarterial chemoembolization with DOX alone [41]and association with other agents [42].These works suggest that DOX has strong antitumor efficacy and still is a promising and safe agent for the treatment of HCC after reduction of toxicity in normal tissues. Recently, with the development of personalized treatment and target chemotherapy for tumor, a number of studies have used over-expressed receptors on the surface of tumor cells as DOX target sites and accordingly designed drug molecules or delivery systems to distribute DOX to tumor cells [43]–[46]. Antibody-conjugated doxorubicin is capable of selectively suppressing tumor growth with limited side effects on normal tissues [47].

Development of ADCs with therapeutic potential involves the optimization of several critical parameters. These include using highly potent drugs that are attenuated and stable while attached to the mAbs, using drug-mAb linkers that allow for the release of active drug only when the mAb has reached the target site, and selecting suitable target antigen [48]. In this study, a human Fab fragment against the human c-Met protein (MetFab) were developed, and conjugated with doxorubicin by acid sensitive hydrazone bond to form an antibody-drug conjugate (MetFab-DOX). The uptake and *in vitro* anti-cancer activity of MetFab-DOX was determined using HCC cell lines. What's more, the localization and anti-tumor activity, and side-effect reduction of doxorubicin *in vivo* was assessed in a nude mouse xenograft model of HCC.

## Materials and Methods

### Ethics Statement

This study was approved by the Ethics Committee of Nanjing Medical University, and all the patients submitted their signed and informed consent to participate. All the animal experiments were approved by the Ethics Committee of Nanjing Medical University, and strictly adhered to the Guiding Principles on the Care and Use of Animals of Nanjing Medical University. At the endpoint, mice were euthanized by an overdose of intraperitoneal pentobarbital and no animal died before euthanasia could take place.

### Cells and agents

The HCC cell lines, including HepG2, Sk-Hep-1, QGY7701, SMMC7721,Bel7402 and mouse embryo fibroblast (NIH3T3), were all purchased from the cell bank of Shanghai Institute of Biochemistry and Cell Biology (Chinese Academy of Sciences, Shanghai, China). Both HepG2 and Sk-Hep-1 were positive for c-Met expression [49]–[51], and NIH3T3 was negative for c-Met expression [52], [53]. All cell lines were cultured in DMEM-H (Invitrogen, Carlsbad, CA, USA) supplemented with 10% fetal bovine serum at 37°C with 5% CO_2_. Doxorubicin was produced by Zhejiang Haizheng Medicine Company (Taizhou, China). The anti-anthrax humanized antibody TEX-IgG which can react with goat anti-human Fab was constructed previously by our lab.

### Screening human Fab fragment against c-Met from phage-display Fab library

A human immunized phage-display Fab library was constructed from peripheral blood lymphocytes from 40 patient volunteers with HCC. This study was approved by the Ethics Committee of Nanjing Medical University, and all the patients submitted their signed and informed consent to participate. Human peripheral blood mononuclear cells were isolated by Ficoll-Pacque gradient centrifugation, and total RNA was prepared by using an RNA Purification kit (QIAGEN, Valencia, CA, USA). For the amplification of Fab gene segments, a unique three-step PCR was used [54]. The resultant Fab was digested with *Sfi*I (Roche Molecular Biochemicals, Mannheim, Germany), and ligated into the phagemid pComb3XSS (provided by the Barbas III Laboratory). After ligation, the recombination phagemid was electro-transformed into *E.coli.*XL1-Blue (Stratagene, La Jolla, CA, USA). After helper phage VCSM13 (Stratagene) was added, the cultures were spun down and phages were precipitated for the phage display antibody library. The library was subjected to seven rounds of panning with S114(c-Met positive cells) and NIH3T3(c-Met negative cells). Specificity of individual phage Fab and soluble Fab were assessed by ELISA. The one, with plasmid of pComB3X-MetFab showed high avidity with c-Met, was sequenced and chosen for the next experiments.

### Production of human Fab fragment against c-Met

The plasmid pComB3X-MetFab was transformed into competent *E.coli.* Top10 F', and the positive insert was identified by PCR amplification of the Fab fragment from bacterial colony. Next, the positive clone was amplified at 37°C for 3 h and induced by 1 M IPTG at 25°C overnight in SB or LB. After centrifugation, the bacterial precipitation was crashed by ultrasonication, and the supernatant was collected and purified by the protein L affinity column. The conditions for the large-scale expression and purification of the human Fab fragment against c-Met were optimized.

### Conjugation of human Fab fragment against c-Met with doxorubicin

**Figure 1:**
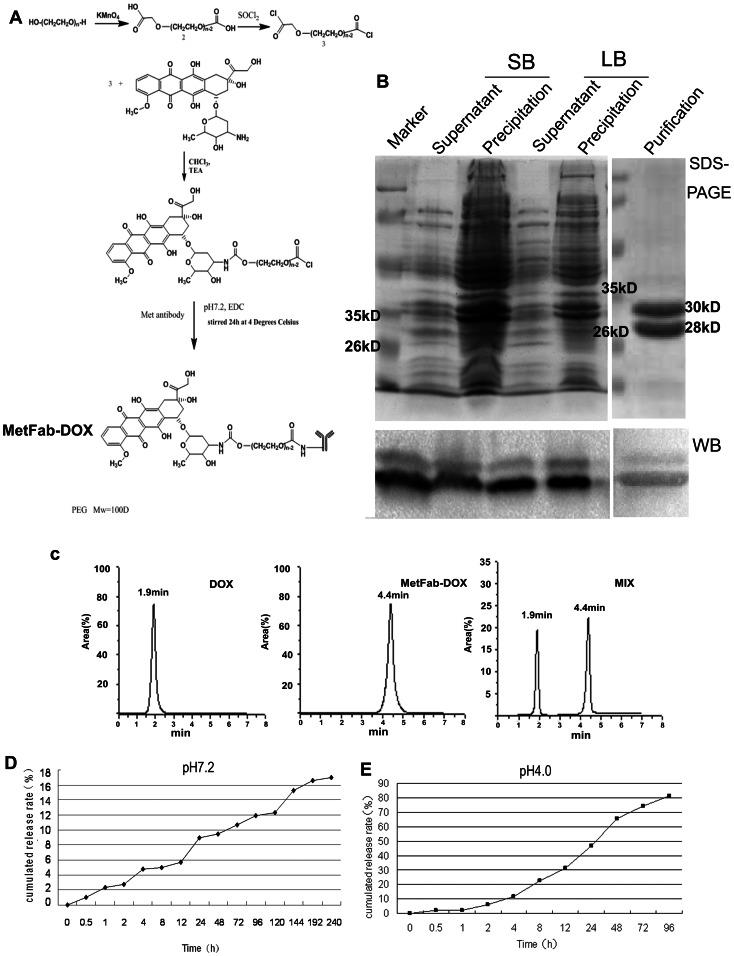
Conjugation of the human Fab fragment against c-Met with doxorubicin. (A)PEG100 was oxidized into compd. **2** in the presence of potassium permanganate, followed by chloridated at 80°C to form compd. **3**. Then PEG-DOX was obtained and condensed with MetFab promoted with EDC by stirring overnight at 4°C. (B)MetFab was expressed in *E.coli.*TOP10F' cultured in LB and SB medium at 25°C, purified by protein L affinity column and identified by Western blot with goat anti-human Fab antibody. The protein was confirmed by Western blot showing the correct molecular size, 28 kDa for κ and 30 kDa for Fd. The sonication supernatant of SB medium contained more expected protein than that of LB medium at 25°C after an overnight induction. (C) MetFab-DOX conjugation was identified by HPLC. A trace of free DOX can be seen at (*t*R) 1.9 min (left). While after reaction, the major peak corresponding to the final conjugate moved at (*t*R) 4.4 min (middle), and no peak of free DOX was observed at (*t*R) 1.9 min. Both peaks can be seen in the admixture of free DOX and MetFab-DOX as control (right). (D)The conjugation was stable in pH7.2 PBS. The amount of cumulated DOX release was about 16.9% after 10 days. (E)The drug released from MetFab-DOX quickly in pH 4.0 PBS, and 81.3% DOX was released within 96 hours.

PEG100 was oxidized into compd. **2** in the presence of potassium permanganate, followed by chloridated at 80°C to form compd. **3** which acylated DOX. Then PEG-DOX was thus obtained. After removing the organic-reaction solvent, the reaction solution was adjusted with PBS at pH 7.2, and then PEG-DOX was condensed with MetFab promoted with EDC by stirring overnight at 4°C (Figure 1A). After concentration by dialysis, MetFab-DOX was purified by poly-dextran gel G100, and the drug loading rate was about 30.4%.

The MetFab-DOX conjugate was examined by HPLC (Waters, Milford, MA, USA) using a reverse-phase column Diamonsil C18 (250 mm×4.6 mm, 5 µm) with UV detection at 254 nm. The mobile phase was comprised of 30% ethanoic acid and 70% acetonitrile at the flow-rate of 1 mL/min.

### *In vitro* release of antibody doxorubicin conjugate

The release rate of DOX from the ADC was measured by a spectrophotometer in triplicate. Briefly, the ADC was suspended in 1 mL of PBS in a filter bag. Then the filter bag was placed in 300 mL medium of PBS (pH 7.2 or pH 4.0) in an orbital shaker at 37°C and shaken at 100 r/min. At predetermined time intervals, 1 mL medium was taken out of the shaker for analysis of DOX concentration, and further analyzed for the accumulated release rate of DOX from the ADC.

### Identification of the c-Met expression in HCC cell lines by Western blot

The expression c-Met in cell lines were performed by Western blot as described [55]. Briefly, the cell lysate was extracted by RIPA solution, and the proteins were separated by 10% SDS-PAGE gel and transferred onto poly(vinylidene difluoride) membrane (Bio-Rad, Hercules, CA, USA). The membrane was blocked with PBS containing 5% non-fat milk at 4°C overnight, incubated with rabbit polyclonal anti-c-Met antibody(Boster Corp., Wuhan, China) at 1∶200 dilution or rabbit polyclonal anti-β-actin antibody (Boster Corp.) at 1∶200 dilution for 1.5 h at RT, washed in PBS with 0.05% Tween 20, and reacted with HRP conjugated goat anti-rabbit IgG (Sigma-Aldrich, St. Louis, MO, USA) at 1∶100 dilution for an additional 1 h at RT. Then the proteins were detected with chemiluminescent substrate as suggested by the manufacturer (Bio-Rad). The ratio of c-Met to β-actin was determined by scanning densitometry using the Quantity One software (version 4.6.3, Bio-Rad), and the expression levels of c-Met was compared among all cell lines.

### Fluorescence-activated cell sorting (FACS) analysis

Cells of each type were grown in the 25 mL cell culture flasks until they reach a confluence of 60%–70%. The cells were trypsinized for obtaining single-cell suspension. Next, 1×10^6^ cells of each type were washed twice with PBS (pH 7.4) containing 1% bovine serum albumin. After blocking with 5% non-fat milk for 30 min, cells were treated with 100 µg/mL MetFab-DOX for 1 h at 4°C. After incubation, the cells were washed three times with PBS followed by FACS analysis with BD Facscalibur using the Cellquest Programme (Beckton Dickinson Biosciences, San Jose, CA, USA).

### Cell ELISA assay

Sk-Hep-1 or HepG2 cells harvested in the logarithmic growth phase were seeded in a 96-well cell culture plate at a cellular density of 2×10^4^ cells/well. Following an overnight incubation, the medium was aspirated, and the cells were washed three times with ice-cold PBS and fixed with 4% paraformaldehyde in PBS for 20 min at room temperature. After removal of the fixing solution and complete drying, the plate was blocked with 1% non-fat milk for 2 h, followed by the addition of 50 µL MetFab, MetFab-DOX and TEX-IgG with the same gradient effective-antibody concentrations from 0.31 µg/mL to 40 µg/mL in triplicate wells. After 2 h, the antibody solution was removed, and the wells were washed six times with 0.5% PBST followed by the addition of goat anti-human Fab coupled to horseradish peroxidase (HRP) antibody at a dilution of 1∶2000 and incubation for 1 h at room temperature. Plates were washed as described above, and visualization was achieved by staining with the TMB solution at room temperature for 30 min and followed by stopping with 1 M H_2_SO_4_. The optical absorbance was measured at 450 nm.

### Immunofluorescence assay *in vitro*

Briefly, Sk-Hep-1 and HepG2 cells were grown in the 96-well cell culture plate at a cellular density of 5×10^3^ cells/well overnight, washed three times with ice-cold PBS, and fixed with 4% paraformaldehyde in PBS for 20 min at room temperature. After blocking with 1% non-fat milk for 2 h, 50 µL MetFab or MetFab-DOX at effective-antibody concentration of 40 µg/mL was added into the wells. Following 4-h incubation, the wells were washed 3 times with 0.5% PBST and the fluorescein isothiocyanate (FITC)-conjugated goat anti-human Fab antibody (Sigma-Aldrich) was added at a dilution of 1∶16. The cells were incubated for 1 h at room temperature. Finally the cells were washed as described above and examined with fluorescence microscopy (Olympus, Tokyo, Japan). NIH3T3 Cells were treated with MetFab-DOX same as the two HCC cells.

### Route of MetFab-DOX transported within HCC cells

Sk-Hep-1 and HepG2 cell lines, at a density of 5×10^3^ cells/well, were seeded onto a 96-well cell culture plate. After overnight incubation, MetFab-DOX and free DOX were added both at 20 µg/mL in terms of the doxorubicin concentration. Following the treatment with MetFab-DOX and free DOX for 1 h, 2 h, 3.5 h and 7 h, the medium was removed, and the cells were carefully washed 3 times with 0.9% saline, and the fluorescent signal of doxorubicin was examined by fluorescence microscopy. NIH3T3 cells were treated and observed same as control.

### Cytotoxicity assay *in vitro*

Cytotoxicity of free DOX and MetFab-DOX was determined by measuring the inhibition of cell growth using a tetrazolium dye (MTT) assay according to a previously established method [28]. Cells harvested in a logarithmic growth phase were seeded in 96-well cell culture plates at a cellular density of 5×10^3^ cells/well. After incubating the cells with various concentrations of free DOX, MetFab-DOX or MetFab for 48 h, the MTT assay was performed and the percentage of cell survive was then determined.

### Anti-tumor therapy in mice bearing HepG2 xenograft

Six-week-old male nude (nu/nu) BALB/c mice (SLRC Laboratory Animal, Shanghai, China) weighing 18–20 g were used for all experiments. The animal experiment was approved by the Ethics Committee of Nanjing Medical University, and was carried out in accordance with the Guiding Principles on the Care and Use of Animals of Nanjing Medical University. All procedures were performed under sodium pentobarbital anesthesia, and all efforts were made to minimize suffering. Tumors were established by a subcutaneous injection of 1×10^6^ HepG2 cells into the back of mice. Tumor volumes were estimated according to the formula: (width)^2^ ×length/2. When tumors reached 100 mm^3^ after about 10 days, the mice were randomly assigned to five groups (8 mice per group) which included control, high-dose DOX, low-dose DOX, MetFab-DOX and MetFab group. Mice received 200 µL i.p. injections of saline, DOX (2 mg/kg), DOX (1 mg/kg), MetFab-DOX (containing 2 mg/kg of equivalent doxorubicin), or MetFab of 4.57 mg/kg (containing an identical antibody concentration as MetFab-DOX injected) every two days. There were a total of 15 injections, and the treatments lasted for 30 days. The sizes of tumors and the body weights were recorded. The mice were sacrificed 2 days after the last injection, and tumors were excised and weighed. In addition, the organs of heart, lung, kidney, spleen and liver were excised and fixed for pathological observation.

### Localization of MetFab-DOX in tumor by immunofluorescence

The mouse HCC model was constructed as above. When tumors reached 500 mm^3^, mice bearing HepG2 xenograft tumors were administered i.v. with DOX (2 mg/kg, *n* = 3) and MetFab-DOX (containing 2 mg/kg equivalent doxorubicin, *n* = 3). Twenty-four hours later, animals were euthanized and tumor tissue was removed, fixed with 4% paraformaldehyde (Sigma-Aldrich) overnight at 4°C and dehydrated by sucrose, followed by snap-freezing and sectioning. To examine the presence of MetFab-DOX, the sections were dried and then blocked with PBS containing 10% bovine serum albumin for 1 h at RT. Fixed tissues were incubated with goat anti-human Fab antibody (1∶500) for 2 h at RT. The slides were washed with PBS and then incubated with anti-goat IgG-FITC-conjugated antibody(1∶100) for another 1 h at RT. Cell nuclei were counterstained by exposure to DAPI (1 µg/mL). After a final wash, the slides were mounted and analyzed under a fluorescence microscope.

### Optical tumor imaging *in vivo*

Fluorescence imaging *in vivo* was performed with a small animal imaging system (Carestream MS FX PRO, Carestream, Rochester, NY, USA). A filter (excitation/emission, 625/700 nm) was used for acquiring fluorescence. The imaging was acquired, overlaid and analyzed by the Carestream MI SE Software (Carestream). Background fluorescence was measured and subtracted by setting up a background measurement. Mice bearing the HepG2 tumor were injected via the tail vein with MetFab-Cy5.5 (2 mg/kg, *n* = 3) or an equivalent dose of Cy5.5 (*n* = 3). The mice without injection were used as a blank control (*n* = 3). Mice were anesthetized with pentobarbital sodium, and images were captured at 3, 6, 12, 24, and 48 h after injection.

### Distribution of MetFab-DOX in a nude mouse xenograft model of HCC

Mice bearing HepG2 xenograft tumors were administered i.v. with DOX (2 mg/kg) and MetFab-DOX (containing 2 mg/kg equivalent doxorubicin), when tumors reached 500 mm^3^. At each selected time point, 3 mice in one group were euthanized by cervical dislocation. Whole blood was collected via cardiac puncture with a heparinized syringe. Heart, liver, spleen, lung, kidney and tumor were dissected out and frozened at −70°C immediately. Plasma was isolated from whole blood by centrifugation at 3000 *g* for 5 min. Tissues homogenateswere prepared in 800 µL water using a Polytron homogenizer (Brinkman Instruments, Mississauga, Ontario, Canada), and 200 µL of H_2_SO_4_ were added to the tissue homogenates. The solutions were then digested for 2 h at 60°C. After the vials cooled to room temperature, 100 µL of AgNO_3_ were added. Then the samples were centrifuged at 12000 *g* for 10 min, and the supernatant were counted in fluorospectrophotometer at excitation wavelength of 500 nm and emission wavelength of 558 nm. The calibration curves for the quantification for DOX were linear over the range of standard concentration between 0.02 and 2.00 µg/mL with a correlation coefficient of *R* ^2^ = 0.9995, and then the concentration of doxorubicin in each tissue was calculated. The plasma was treated similarly as tissue homogenates, 50 µL H_2_SO_4_ and 25 µL AgNO_3_ were added to 200 µL plasma samples and treated as above.

### Data analysis

All the quantitative data were described as means ± standard deviation, and analyzed by Variance Analysis and SNK-*q* test of SPSS 13.0. Differences were considered significant at *P*<0.05 (two-tailed).

## Results

### Screening plasmids containing human Fab fragment against c-Met

A phage display antibody library was constructed with a capacity of 2.0×10^9^. After seven rounds of panning, 95 individual phage clones were selected randomly and amplified to test for specific binding to c-Met protein by phage ELISA. Twenty-two clones were found to react specifically with the c-Met protein by phage ELISA (data not shown). Only one unique DNA sequences were found in the binding clones. The plasmid was sequenced and chosen for large-scale expression and purification.

### Production of the MetFab with optimal parameters

The optimal parameters for the production of human Fab fragment against c-Met were obtained after testing the type of culture medium, the induction temperature, and the concentration of IPTG. For example, as shown in Figure 1B, the sonication supernatant of SB medium contained more expected protein than that of LB medium at 25°C after an overnight induction. Finally, the optimal Fab antibody production was carried out by using the SB culture medium, 25°C induction temperature, and 1 M IPTG. The expected protein was purified from the sonication supernatant with high purity by protein L affinity column. And the final yield reached 5 mg per liter liquid of bacteria.

### Identification of MetFab conjugated with doxorubicin

Identification of MetFab conjugated with doxorubicin by HPLC was according to the signal of DOX under 254 nm. The elution pattern of the DOX and MetFab-DOX was reported in Figure 1C. A trace of free DOX can be seen at (*t*R) 1.9 min. While after reaction, the major peak corresponding to the final conjugate moved at (*t*R) 4.4 min, and almost no peak of free DOX was observed at (*t*R) 1.9 min. It is because the molecular weight of MetFab-DOX was much higher than DOX. And from the delay of the peak time, we can consider that the molecule of chemical synthesis product (MetFab-DOX) was different from that of the conventional DOX. Both MetFab and DOX were conjugated completely because one peak was observed, while two peaks can be seen in the admixture of free DOX and MetFab-DOX as control.

### Drug release pattern changes of MetFab-DOX under neutral or acid condition

*In vitro* DOX release profiles from MetFab-DOX in PBS pH7.2 are shown in Figure 1D. The drug release behavior from the conjugate was slow in pH7.2 PBS, and the amount of cumulated DOX release over 10 days was about 16.9%. While in pH 4.0 PBS (Figure 1E), the DOX was released more quickly than in pH7.2 PBS, and 81.3% DOX was released within 96 hours. It is mainly due to that the MetFab-DOX can be hydrolyzed under pH 4.0 as the study designing.

### c-Met expression in HCC cell lines

**Figure 2:**
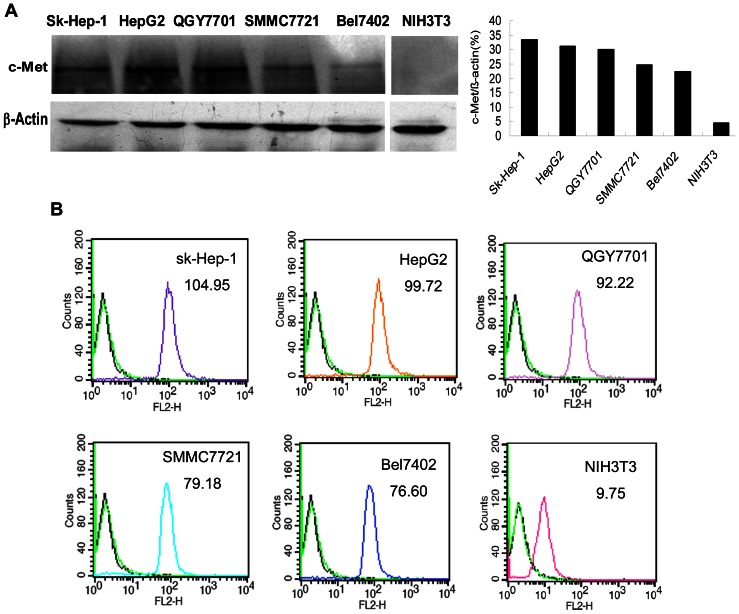
The c-Met expression in each cell line was detected by Western Blot using cell lysate(A) and the binding efficacy of MetFab-DOX on different cells was detected by FACS(B). (A)All HCC cell lines expressed c-Met protein, while no expression of c-Met was observed in NIH3T3 cells. (B) The c-Met expression level was presented as the value of fluorescence intensity by FACS. The number in the upper right was the average fluorescence intensity of each cell lines. Primary antibodies were as follow: no primary (blank line), nor-related control antibody-PE (green line) and MetFab-DOX (colorful line). The result of FACS was conformed to the c-Met expression by WB.

As shown in Figure 2A, most HCC cell lines tested here express c-Met protein with 140 kDa. While there is no c-Met protein expression in NIH3T3 cells, the same as reported by other researches. In HCC cell lines, HepG2 and Sk-Hep-1 had high expression of c-Met, when compared the relative expression ratio to β-actin.

In the FACS assay, the fluorescent signal intensities varied among different cell lines probably own to the fact that different cell lines have various c-Met expression levels on the cell membrane resulting in various binding abilities of MetFab-DOX to cancer cells. NIH3T3 cells which do not express c-Met demonstrated almost no fluorescence. Furthermore, the result demonstrated that the MetFab-DOX was capable of specifically recognizing and binding the c-Met protein in live cells with various c-Met expression levels (Figure 2B).

### MetFab-DOX can bind HCC cells with c-Met expression similar as MetFab

**Figure 3:**
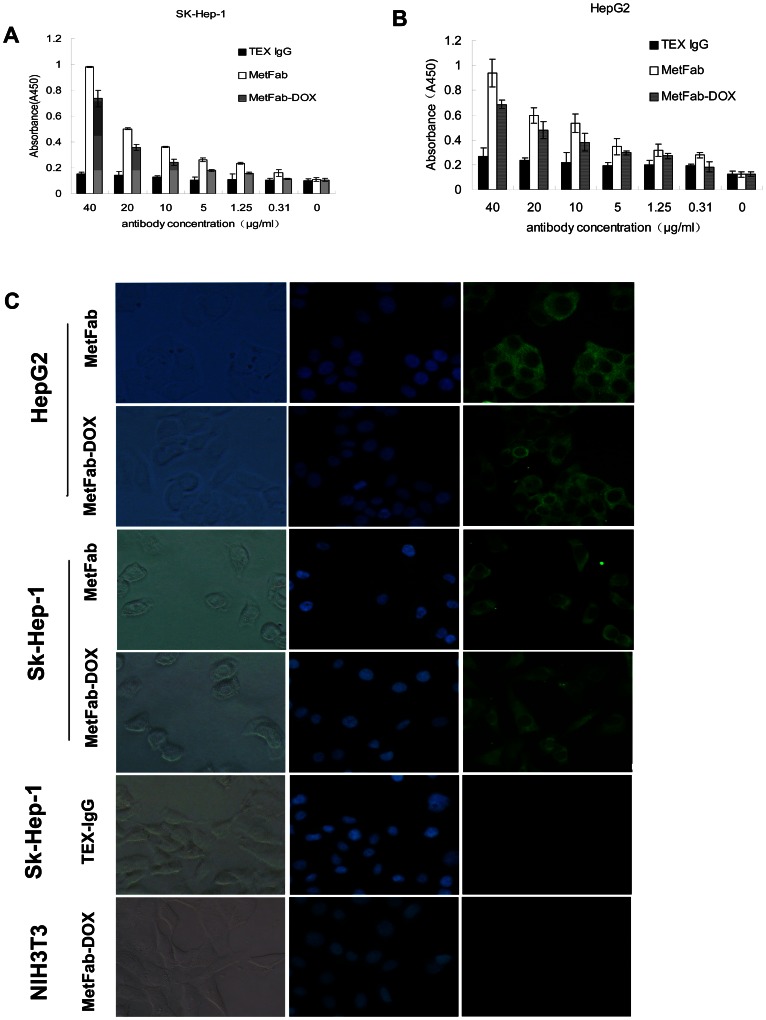
Binding efficacy of MetFab and MetFab-DOX on different cells was detected by ELISA (A, B) and immunofluorescence assay(C). Sk-Hep-1(A) and HepG2 (B) were incubated with MetFab, MetFab-DOX and TEX-IgG at antibody concentration (40, 20, 10, 5, 1.25, and 0.31 µg/mL). Both MetFab and MetFab-DOX can bind Sk-Hep-1 and HepG2 cells specifically, but TEX-IgG can't even at 40 µg/mL. The absorbance values of 3 groups were analyzed by Variance Analysis, and there were significant differences among 3 groups. Furthermore, using SNK- *q* test, there were significant difference between TEX-IgG group and MetFab group (*P*<0.05) or MetFab-DOX group (*P*<0.05), while no significant difference between MetFab group and MetFab-DOX group. (C)The binding efficacy of MetFab and MetFab-DOX was assessed by immunofluorescence observation. Both Sk-Hep-1 and HepG2 cells have green fluorescence with MetFab or MetFab-DOX. No signal was observed in Sk-Hep-1 cells incubated with TEX-IgG and in NIH3T3 cells incubated with MetFab-DOX.

As shown in cell ELISA results (Figure 3A and 3B), MetFab-DOX and MetFab could specifically bind the c-Met on the Sk-Hep-1 or HepG2 cell surface in a dose-dependent manner. While the anti-anthrax antibody TEX-IgG that can react with HRP-conjugated goat anti-human Fab antibody could not bind to the Sk-Hep-1 or HepG2 cells even at a high antibody concentration. There were significant differences in absorbance values between TEX-IgG group and MetFab group (*P*<0.05) or MetFab-DOX group (*P*<0.05). By comparing the optical absorbance values of MetFab-DOX and MetFab, it could be concluded that the affinity of MetFab-DOX was slightly lower compared to its parental MetFab antibody. However there was no significant difference in absorbance values between MetFab and MetFab-DOX groups (*P*>0.05). Taken together, these results demonstrated that MetFab-DOX can bind the c-Met-positive cells similarly as MetFab.

In the immunofluorescence assay *in vitro* (Figure 3C), both MetFab-DOX and MetFab could specifically bind the c-Met expressing-Sk-Hep-1 and HepG2 cells according to the immunofluorescence microscopic observation. The cells treated with these two antibodies exhibited green fluorescence around the cell surface, because both of them could be recognized by FITC-conjugated goat anti-human Fab. While no signal was observed in Sk-Hep-1 cells incubated with TEX-IgG and in NIH3T3 cells incubated with MetFab-DOX. These results implied that the binding avidity of MetFab-DOX to the cell surface epitope was not affected substantially after the chemical conjugation.

### Route of MetFab-DOX transported within HCC cells was different from that of DOX

**Figure 4:**
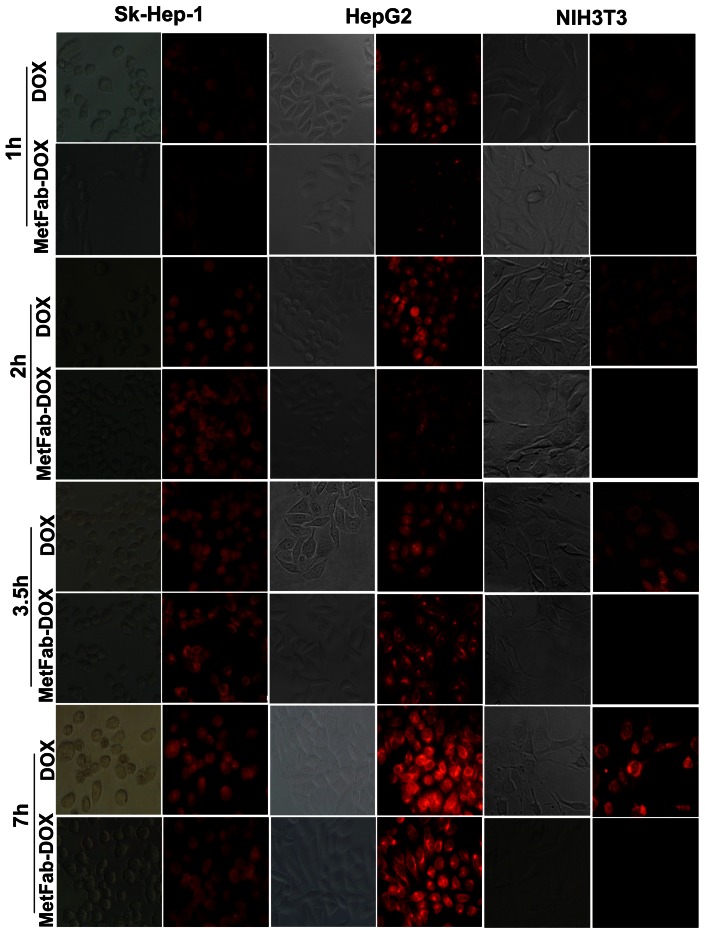
The drug transport pathway was observed by fluorescence microscopy. Sk-Hep-1, HepG2 cells and NIH3T3 cells were incubated with free DOX and MetFab-DOX for 1, 2, 3.5, 7 h. The red fluorescent signal could be detected quickly in the nucleus of cells after incubation with free DOX. In contrast, the MetFab-DOX was clearly distributed in the membrane, cytoplasm and perinuclear zone after incubation for 2 hours, and with prolonged time, the signal of MetFab-DOX can be seen in the nucleus. The conjugate MetFab-DOX can't enter the NIH3T3 cells even after 7 h, and no red signal can be seen, not as free DOX. (Magnification, ×400).

In order to observe how doxorubicin was transported by MetFab into the cells with high expression of c-Met and compare MetFab-DOX and free DOX in their ability to be internalized, Sk-Hep-1 and HepG2 cells were incubated with MetFab-DOX and free DOX for various time points. As shown in Figure 4, after 1 h of treatment, a few red sparkles could be seen in the cells treated with free DOX, whereas a few weak red fluorescent spots could be observed on the surface of cells treated with MetFab-DOX. After 2 h, more red sparkles especially in the cellular nuclei could be identified in the cells treated with free DOX than before. While at this time point, stronger red fluorescence could be observed in the membrane of cells treated with MetFab-DOX than before. And 3.5 h later, the increased red fluorescence could be seen in the nuclei of cells treated with free DOX, and on the surface and in the cytoplasm of the cells treated with MetFab-DOX. Finally at 7 h, cells treated with MetFab-DOX showed significant fluorescence distributed on the surface, and in the cytoplasm and nucleus of Sk-Hep-1 and HepG2 cells.

NIH3T3 cells which do not express c-Met exhibited red fluorescence in the nuclei following treatment with free DOX after 1 h of treatment, and the red signal became strong with time prolonging, but no staining was observed following treatment with MetFab-DOX even for 7 h.

### MetFab-DOX had cytotoxicity effect on HCC cell lines *in vitro*

**Figure 5:**
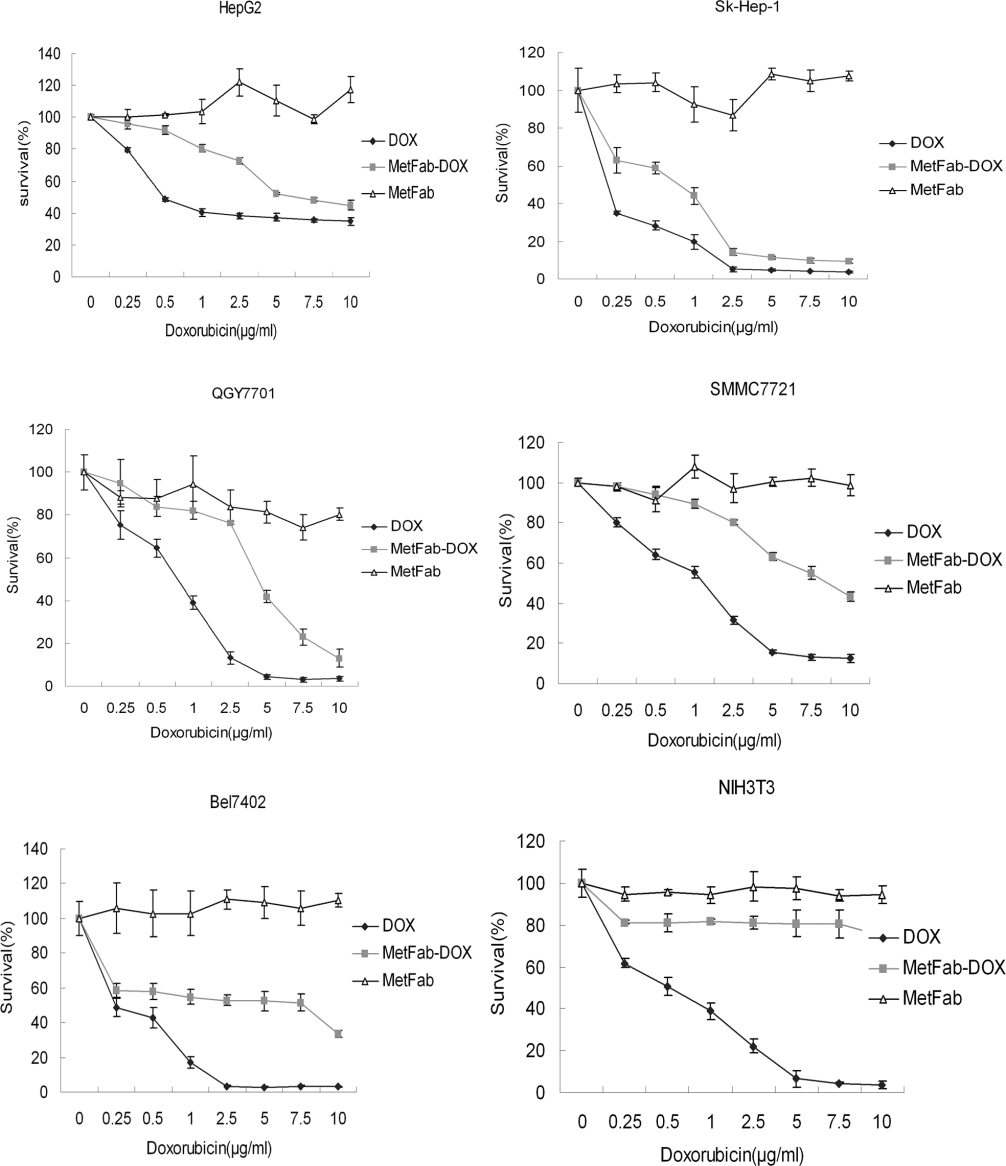
Anti-tumor cytotoxicity *in vitro* of MetFab-DOX versus free DOX on different cells. Viability was assessed with MTT assay in HCC cells at 48 h after treatment with free DOX, MetFab-DOX and MetFab, respectively. In the experiment, the equivalent doxorubicin concentrations in the free DOX and MetFab-DOX were 10, 7.5, 5, 2.5, 1, 0.5 or 0.25 µg/mL, while the MetFab had an equivalent antibody concentration as MetFab-DOX, which was 22.86, 17.14, 11.43, 5.71, 2.29, 1.14, 0.57 µg/mL. In all those cell lines screened, no visible cytotoxicity was found in cells treated with MetFab only. There were significant differences in survival rate of HCC cells between MetFab group and MetFab-DOX group (*P*<0.05) or DOX group (*P*<0.05). Furthermore, there were significant differences in the survival rate of HCC cells between MetFab group and MetFab-DOX group (*P*<0.05). However, in NIH3T3 cells, there were significant differences between DOX group and MetFab group (*P*<0.05). In contrast, there was no significant difference between MetFab-DOX group and MetFab group (*P*>0.05). Results were confirmed with duplicate experiments. The survival rates of treated samples were normalized to the untreated controls.

In all those cell lines screened, no visible cytotoxicity was found in cells treated with MetFab only. There were significant differences in survival rate of HCC cells between MetFab group and MetFab-DOX group (*P*<0.05) or DOX group (*P*<0.05), and it is implied that both MetFab-DOX and free DOX showed a potent and dose-dependent cytotoxicity effect on the HCC cells (Figure 5). Furthermore, the survival rate of HCC cells in DOX group was significantly different from that in MetFab-DOX group(*P*<0.05), and it is implied that the cytotoxicity of free DOX was higher than that of MetFab-DOX on those HCC cell lines based on survival rate of cells after treatment for 48 h. In NIH3T3 cells, a similar trend in the cytotoxicity was observed in cells treated with free DOX, and there were significant differences in survival rate between DOX group and MetFab gtroup(*P*<0.05). In contrast, little cytotoxicity was observed in NIH3T3 cells treated with MetFab-DOX under the same experimental conditions, and there was no significant difference in survival rate between MetFab-DOX group and MetFab group (*P*>0.05).

We also compared the IC_50_ of MetFab-DOX and DOX *in vitro* for different cell lines. The cell sensitivity to doxorubicin decreased when cells were treated with MetFab-DOX. But the reduced degree of sensitivity was various in different cell lines. We use the fluorescence intensity of c-Met detected by FACS analysis to represent c-Met expression levels of cells, in order to study a potential correlation between the expression of c-Met and the potency of MetFab-DOX. As shown in Table 1, according the concentration of effective-doxorubicin, the ratio of IC_50(MetFab-DOX)_ to IC_50(DOX)_ was 4.8 to 6.0 in HepG2, SK-Hep-1 and QGY7701 cells with high expression of c-Met, while about 10 in SMMC7721 and Bel7402 cells with middle expression of c-Met. In NIH3T3 cells with no expression of c-Met, IC_50(DOX)_ was 0.504 µg/ml, while the IC_50(MetFab-DOX)_ can't be calculated because MetFab-DOX almost has no cytotoxicity effect on NIH3T3. It suggested that the anti-tumor effects of MetFab-DOX are immunological specific and have some correlation with c-Met expression in cell surface.

**Table 1:** The IC_50_ of doxorubicin in cells treated with DOX or MetFab-DOX with equivalent effective-concentration of doxorubicin.

	DOX( µg/ml)		MetFab-DOX ( µg/ml)			
Cell line	IC_50_	95%CI	IC_50_	95%CI	Ratio of IC_50_	c-Met* expression
HepG2	1.136	0.036–3.736	6.817	5.465–9.091	6.00	99.72
Sk-Hep-1	0.112	0.052–0.184	0.579	0.347–0.839	5.17	104.95
QGY7701	0.68	0.571–0.797	3.263	2.010–5.754	4.80	92.22
SMMC7721	1.072	0.872–1.294	8.783	6.968–11.800	8.19	79.18
Bel7402	0.258	0.117–0.413	2.854	1.168–11.392	11.06	76.60
NIH3T3	0.504	0.388–0.628	>8345826353.00	-	-	9.75

* :The c-Met expression level was presented as the value of fluorescence intensity by FACS. IC_50(MetFab- DOX):_ The IC_50_ value of MetFab-DOX to cells after 48 h treatment. IC_50(DOX)_. The IC_50_ value of DOX to cells after 48 h treatment. Ratio of IC_50:_ IC_50(MetFab- DOX)_/IC_50(DOX)._

Furthermore, we study a potential correlation among c-Met expression levels, DOX potency and MetFab-DOX potency through Spearman's analysis. A lack of significant correlations between c-Met expression levels and DOX potency was noticed (*r* = 0.268, *P* = 0.561). While a significant correlations between c-Met expression levels and MetFab-DOX potency was found (*r* = −0.777, *P* = 0.040). Combined, these findings suggest that c-Met expression levels could help to predict the sensitivities of the various HCC cells toward MetFab-DOX, if the cells had comparable sensitivity to DOX.

### MetFab-DOX had anti-tumor effect in mice bearing HepG2 xenograft with reduced side effect

**Figure 6:**
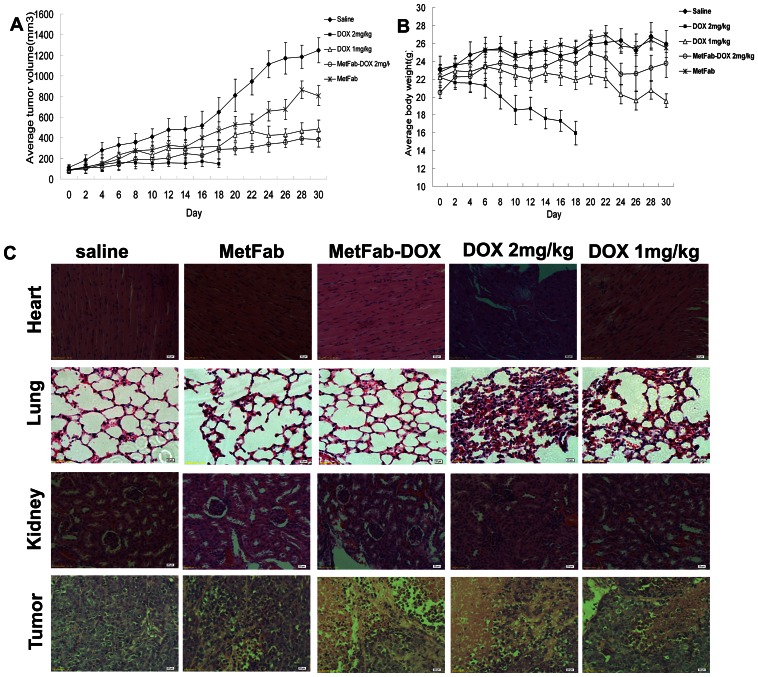
Tumor inhibition assay in mice bearing HepG2 xenograft. (A, B): Subcutaneous tumors were established in mice inoculated with HepG2 cells. Animals were given i.p. injection of DOX and MetFab-DOX at the indicated doses every two days for 15 injections, except that the mice treated with 2 mg/kg DOX died after 10th injection. The mice injected with saline were as control, and the MetFab was given at the same antibody concentration as MetFab-DOX. The average tumor volumes (A) and mice body weight (B) were determined from 8 animals per group. (A) There were significant differences in tumor growth among groups (*P*<0.001). The growth of tumors was inhibited by free DOX, MetFab-DOX and MetFab when compared with saline (*P*<0.001). There was no significant difference in tumor volume between the MetFab-DOX and high-dose DOX treatments with equivalent doxorubicin dosage (*P* = 0.08). (B) There were significant differences in body weight between groups treated with DOX and any other reagents (*P*<0.001). High-dose or low-dose DOX treatment resulted in a significant reduction of body weight compared to MetFab-DOX treatment (*P*<0.001). There was no significant difference in body weight between MetFab and saline treatment. (C)The tissue pathological observation of nude mice bearing HepG2 cells after different treatments. All the tissue sections were prepared by HE staining. There were significant pathological changes in heart, lung and kidney in mice treated with free DOX. And there was inapparent necrosis in tumor tissue of saline group, while profound necrosis and bleeding in tumors of DOX and MetFab-DOX group.

After the treatment, the growth of tumors was inhibited by free DOX, MetFab-DOX and MetFab when compared with saline (*P*<0.001, Figure 6A). At the end of the experiment, the tumor inhibition ratios following various treatments were calculated to be 90.38% for high-dose DOX, 59.67% for low-dose DOX, 65.40% for MetFab-DOX, and only 30.80% for MetFab. There was no significant difference in tumor volume between the MetFab-DOX and high-dose DOX treatments with equivalent doxorubicin dosage (*P* = 0.08).

At the same time, the body weights of mice were measured to evaluate the *in vivo* toxicity of DOX (Figure 6B). At the end of experiment, the average body weight was 15.95±1.32 g in high-dose DOX treatment group, 19.56 ±0.77 g in low-dose DOX treatment group, 23.78±1.57 g in MetFab-DOX treatment group, 25.91±1.50 g in saline control group and 25.52±0.51 g in MetFab treatment group. There were significant differences in body weight between groups treated with DOX and any other reagents (*P*<0.001). High-dose or low dose DOX treatment resulted in a significant reduction of body weight compared to MetFab-DOX (*P*<0.001). Furthermore, after 10 times of injection, 3 mice died after the high-dose DOX treatment, and the left 5 mice died in the following 4 days, while no death was observed in the MetFab-DOX group until the end of experiment. In conclusion, DOX had significant side effects irrespective of the dose used, whereas the antibody-doxorubicin conjugation significantly reduced the toxicity of chemotherapy.

In tissue pathology examination (Figure 6C), it is found that the myocardial filaments were well organized, smooth and tightly packed. However, the organization of myocardial filaments in the DOX treated mice was disrupted, and vacuolization was evident. In contrast, there was no significant disruption of the myocardial filaments and vacuolization in the MetFab-DOX treated mice when compared with that of control. MetFab itself did not induce any pathological damage in the heart tissue. Severe hyperemia and hemorrhage in the lung alveolar space and extensive atrophy of kidney glomerulus were identified in DOX-treated mice, which demonstrated pathology changes secondary to heart failure. Furthermore, profound necrosis in the tumor tissues in mice treated with DOX or MetFab-DOX was observed compared to MetFab only or saline. No significant pathological changes in liver and spleen were observed in any mice examined.

Although there was no significant difference in tumor inhibition between the MetFab-DOX and DOX treatments with equivalent doxorubicin dosage, MetFab-DOX treatment significantly attenuated manifestation of the side effects induced by DOX.

### MetFab-DOX can localize in tumor tissue

**Figure 7:**
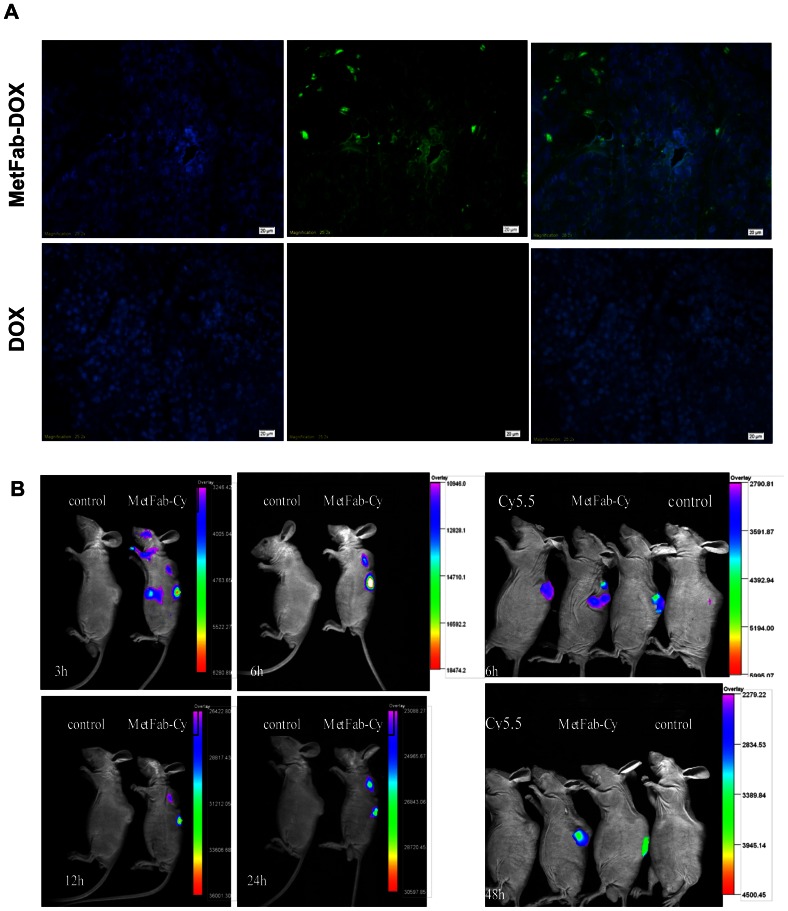
The localization of conjugate was confirmed both by (A)immunofluorescence staining of frozen tumor tissue sections *in vitro* and (B)optical tumor imaging *in vivo.* (A)Mice bearing HepG2 xenograft was injected i.v. with MetFab-DOX and free DOX, the tumor tissue was removed 24 h later and observed after incubated with goat anti-human Fab and FITC-anti-goat IgG. No obvious signal can be found in the tumor tissues from mice treated with DOX as control, while the green fluorescence was observed in mice treated with MetFab-DOX. (B)Mice bearing HepG2 xenograft was injected i.v. with MetFab-Cy5.5 and observed under small animal imaging system. Mice without injection were as blank and mice with cy5.5 injection as control. The fluorescent signal detected at the tumor site reached the highest fluorescent intensity at 12 h post-injection of MetFab-DOX (left). The fluorescent signal in Cy5.5-treated mice disappeared quickly, and could not be observed at 48 h post-injection. In contrast, the fluorescent signal in the MetFab-Cy5.5 group was still observed in tumor tissue at 48 h post-injection (right).

The MetFab-DOX or DOX were injected into mice bearing HepG2 xenograft tumors, and the tumors were harvested 24 h later. Tumor sections were made and assayed for the presence of MetFab-DOX by immunofluorescence using an anti-human Fab antibody. As shown in Figure 7A, tumors obtained from the MetFab-DOX-injected mice showed green fluorescence with anti-human Fab antibody, while there was no green fluorescence signal observed in the tumor of the DOX-treated group. It demonstrated that MetFab-DOX can localize in the tumor tissue at 24 h after injection. Besides, the localization of the MetFab-DOX corresponded to regions around the vasculature. The reason could be that the cells around the vasculature can get much MetFab-DOX easier than those away from vasculature did.

Figure 7B shows typical NIR images of mice bearing HepG2 tumors 3 h, 6 h, 12 h, and 24 h after i.v. injection of MetFab-Cy5.5. No auto-fluorescence was detected from the mouse as blank. The fluorescent signal could be significantly detected at the tumor site at 3 h post-injection, becoming stronger with prolonged time, and reaching the highest fluorescent intensity at 12 h post-injection. Meanwhile, the fluorescent signal in kidney was observed after injection, and was attenuated obviously at 24 h post-injection, while the fluorescent signal was still strong at tumor site at this time point. Compared with blank control, the fluorescent signal in Cy5.5-treated mice disappeared quickly, and could not be observed at 48 h post-injection. In contrast, the fluorescent signal in the MetFab-Cy5.5 group was still observed in tumor at 48 h post-injection.

### MetFab-DOX distribution was different from DOX in a nude mouse xenograft model of HCC

**Figure 8:**
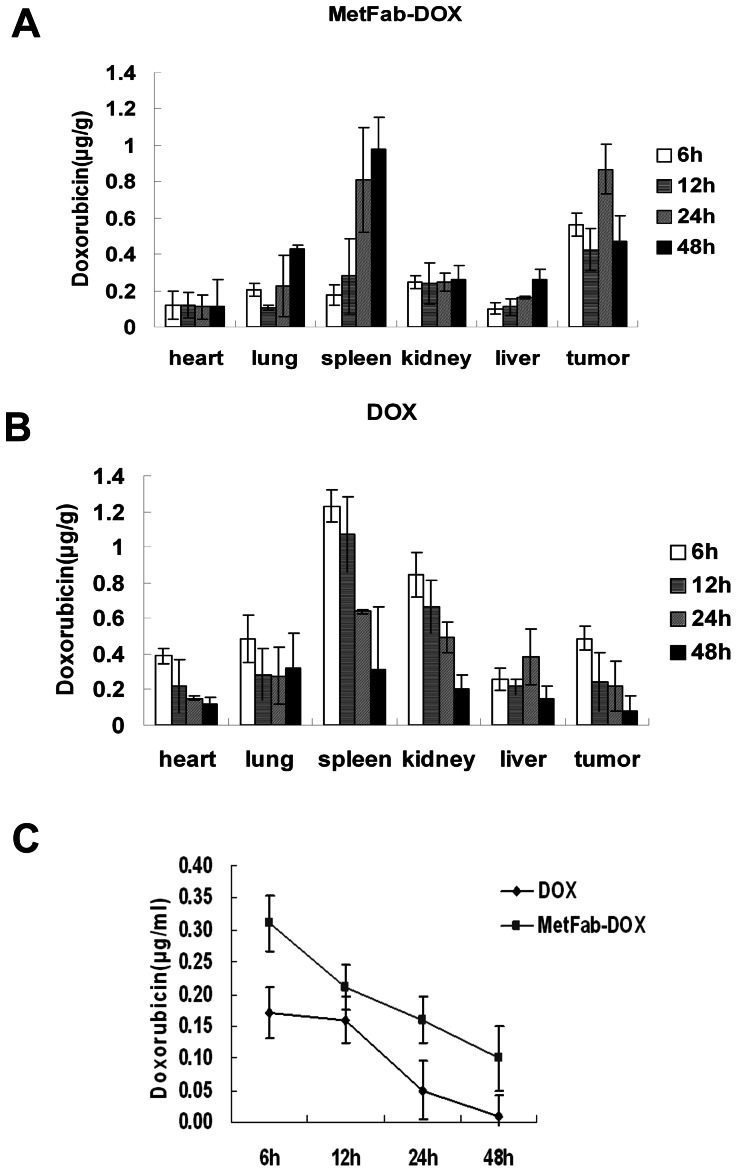
The doxorubicin distribution in tissues of mice after injection of DOX or MetFab- DOX with equivalent doxorubicin dose. The concentration of doxorubicin in tumor tissue at each time point was higher after injection of MetFab-DOX (A) than after injection of conventional DOX(B)(*P*<0.05). On the contrary, after 6 h, 12 h and 24 h injection, a higher concentration of doxorubicin was significantly present in cardiac or kidney tissue after administration of conventional DOX (B) than after administration of MetFab-DOX (A)(*P*<0.05). Although the peak time was prolonged in lung, spleen and liver of MetFab-DOX group when compared with DOX group, there were significant differences in spleen between two groups(*P* = 0.024), while no significant difference was found in liver and lung between two groups(*P*>0.05). (C) The concentration of doxorubicin in plasma at each time point was higher after injection of MetFab-DOX than after injection of conventional DOX (*P*<0.05).

The concentrations of doxorubicin in tumor tissues at each time point were significantly higher after injection of MetFab-DOX than after injection of an equivalent amount of doxorubicin (*P* = 0.038, Figure 8A, 8B). This was reversed in heart and kidney tissue. At 6 h,12 h and 24 h after injection, a higher concentration of doxorubicin was significantly present in cardiac(*P* = 0.038) and renal (*P* = 0.001) tissue after administration of conventional doxorubicin than after administration of MetFab-DOX with an equivalent doxorubicin dose. Additionally, the trend of DOX concentration was different in spleen between conventional doxorubicin and MetFab-DOX groups, increasing with time prolonged in MetFab-DOX group while decreasing in conventional doxorubicin group. The reason could be that the spleen is the largest unit of reticuloendothelial system(RES), and the MetFab-DOX comprised of foreign protein(human antibody fragment) could be engulfed and blocked by RES. Accordingly, the doxorubicin concentrations in the spleen of MetFab-DOX group was different form those of conventional doxorubicin group, and there were significant differences between two groups (*P* = 0.024). Similarly, the trend of DOX concentration in the liver was increased with time prolonged in MetFab-DOX group while decreased in conventional doxorubicin group, but the differences between two groups were not significant (*P*>0.05). In the MetFab-DOX group, the concentrations of doxorubicin were lower in liver than in tumor, which implied that if the tumor was growing in the liver tissue, the normal liver cells could receive much less doxorubicin than tumor cells when MetFab-DOX was used for target chemotherapy. There also was some differences in distribution of doxorubicin in lung but without significance (*P*>0.05).

The plasma doxorubicin concentrations of mice treated with conventional doxorubicin were significantly lower than those treated with MetFab-DOX at each time point detected (*P* = 0.014, Figure 8C). These data indicate that MetFab-DOX is a targeted ‘prodrug’ that can selectively deliver more doxorubicin to c-Met-expression tumor tissue than conventional doxorubicin.

## Discussion

In this study, we show that a novel ADC (MetFab-DOX) consisting of doxorubicin conjugated to a human anti-c-Met Fab can target to c-Met expression HCC cells and still preserve the cytotoxicity effect of DOX after the conjugation, and it only specifically bound to HCC cells that expressed c-Met and had cytotoxic effect on those cells, but not on the cells that did not express c-Met. Furthermore, MetFab-DOX could significantly reduce the DOX-induced toxicity to heart, kidney and lung as well as its effect on body weight loss, with a similar anti-tumor effect as free DOX in mice model. In addition, the concentrations of doxorubicin in tumor tissue and plasma at each time point after injection of MetFab-DOX were higher than that after injection of conventional doxorubicin, but the results were verse in heart and kidney. This could probably effectively reduce the side effect of doxorubicin in treating HCC, which was confirmed by the experiment *in vivo* of HepG2-bearing nude mice, supporting that the MetFab-DOX may have therapeutic potential for treating HCC and reduce the side-effect of doxorubicin.

Development of ADCs with therapeutic potential involves the optimization of several critical parameters[56], [57]. One key factor of ADCs is the choice of target antigen for antibody. The target antigen should internalize upon mAb binding, have high expression on tumor cells, and little to no expression on normal cells. In this study, c-Met was chosen as the target antigen for ADCs for several considerations. Firstly, c-Met is expressed on the majority of human HCC. In our experiments, almost all HCC cell lines expressed c-Met at various levels. Secondly, our results confirmed that the MetFab could specifically bind c-Met on the cell surface and be internalized with DOX efficiently. Thirdly, although c-Met is involved in organ regeneration, as demonstrated for liver and kidney, embryogenesis, hematopoiesis, muscle development, and in the regulation of migration and adhesion of normally activated B cells and monocytes, imaging studies using a radiolabeled version of anti-c-Met antibody revealed preferential distribution to tumors in mouse model with xenografts, suggesting that targeting of the immunoconjugate to normal mouse tissues is minimal [58]. Furthermore, our study also confirmed that ADC targeted c-Met can effectively delivery more drugs to the tumor tissue than conventional doxorubicin did, but less drugs to heart and kidney tissues than conventional doxorubicin. Thus, c-Met is suitable for ADC target, and the anti-c-Met antibody, MetFab, can be vectors for selectively delivering the drugs to HCC tissues.

The linker for the ADCs also is a very important factor which may have an impact on the efficiency of the drug. The linker is important to maintaining the stability of the ADCs, and allow for the release of active drug only when the mAb has reached the target site [59]–[61]. MetFab-DOX is produced by chemical synthesis, which contains an acid sensitive hydrazone bond that is relatively stable in pH7.2 PBS, while can release drug at pH4.2, which demonstrates that the conjugate is stable and does not release drug in plasma before get to the tumor tissue, but allows DOX to be intracellularly released from the antibody vector in the endosomal or lysomal compartment after pH change. In addition, DOX and MetFab are not linked directly but through a bridging molecule of PEG, which maximally guarantees the function of DOX and MetFab. Then we detected the binding efficiency of the ADC (MetFab-DOX) via chemical synthesis. In FACS assay, MetFab-DOX can bind different cells with various c-Met expression levels, and the binding efficacy was correlated with c-Met expression levels on the cell membrane, conformed to the result of Western blot assay. ELISA analysis and immunofluorescence observation showed that MetFab-DOX in fact had a similar specific binding affinity as MetFab. All the above results imply that the chemical conjugation process we taken does not compromise the biologic activity of MetFab in binding the target cells.

As we all know, it is very important for ADCs development by using highly potent drugs. Most successful ADCs used highly toxic agents that can not be common used in the clinic, for example, Auristatins, Maytansines and Calicheamicins. The use DOX may be not the best choice, although it has been widely used in HCC chemotherapy. But the DOX has some advantages for ADC research. First, it has high water-solubility that makes the conjugation process simply. Second, it has red fluorescent signal that can be easily observed after excitation under fluorescence microscope, and the route of ADC transferring into the live cells can be observed directly. Furthermore, the drug concentration in the tissues or blood can be conveniently detected by fluorospectrophotometer according to the fluorescent signal of DOX. As a result, we consider that DOX still is a good model drug for some target therapy research.

By fluorescence microscopy, red fluorescent signal could be detected quickly in the nucleus of cells after incubation with free DOX, no matter the c-Met expression level of cells, because free DOX was transported into cells by a passive diffusion mechanism and bound nucleus rapidly. In contrast, the MetFab-DOX was clearly distributed in the membrane, cytoplasm and perinuclear zone after incubation for 2 hours. With prolonged time, MetFab-DOX may be hydrolyzed to release the active DOX in the cytoplasm which then penetrates the nuclear membrane, and all those existed in HCC cells with c-Met expression but not in NIH3T3 cells without c-Met expression. On the other hand, it suggests that at the same time point the active DOX released from the conjugate and partitioned to the nucleus was lower than that from free DOX. Therefore, its *in vitro* cytotoxicity was not as high as that of free DOX, and 48 hours of incubation seemed to be an insufficient period for the release of DOX from the conjugate, which is consistent with many other reports [16], [62]. Although we can't get the conclusion that MetFab-DOX was more powerful than conventional DO\ in cytotoxity effect on HCC cells *in vitro*, it doesn't mean that the cytotoxicity effect of MetFab-DOX was less than conventional DOX. While with time prolonged, the bioavailable drug from MetFab-DOX could be increased and comparable with that from conventional DOX, and the cytotoxicity effect could be comparable between MetFab-DOX and conventional DOX. But because of the limit of cell culture *in vitro,* it is difficult to evaluate cell viability accurately when the culture time was prolonged. Besides, the target and enrich effect of MetFab-DOX to the specific tumor cells can't be observed *in vitro.* As a result, it is necessary for us to detect the real anti-tumor effect of MetFab-DOX *in vivo*.

Additionally, our study had several potential limitations. First, although we found that MetFab-DOX could inhibit the tumor growth in HCC tumor with c-Met expression model, we have not evaluated the actual effect of the ADC against human c-Met-negative cells as well as nonneoplastic c-Met-positive cells as control. Second, although one dose of MetFab-DOX was used and have effect in preclinical mice model experiments, a potential therapeutic window should be completed in the future. Unexpected toxicities may be identified if the dose of MetFab-DOX rose, especially to c-Met-expressing normal tissues or cells. Third, the subcutaneous transplanted tumor model was used for evaluating the treatment efficiency of MetFab-DOX. If the orthotopic transplantation tumor model was used, the anti-tumor effect by MetFab-DOX could be better *in vivo*, because the orthotopic transplantation tumor has more sufficient blood supply for ADCs transportation than subcutaneous transplanted tumor. Fourth, although the human anti-c-Met Fab used in our study could diminish the potential response of heterogenetic antibodies, while it may reduce antibody avidity, and the strategies to overcome the problem were needed in the future, such as carrying out affinity maturation of Fab, converting Fab to full-molecular antibody by genetic recombination, and so on.

In summary, the findings herein provide a novel c-Met-targeted ADC with potent antitumor activity in HCC with limited side effects, and this study provided a basis for the future research. However, a lot of work appears to be warranted, for example, increasing the anti-tumor efficiency of the ADC, examining the pharmacokinetic study of the ADC, establishing optimal concentrations and dosages for potential clinical applications, and even changing the conjugated drug to some highly toxic agents fitting ADC development for target therapy of HCC.
